# Economic burden of malaria on rural households in Gwanda district, Zimbabwe

**DOI:** 10.4102/phcfm.v9i1.1317

**Published:** 2017-08-28

**Authors:** Resign Gunda, Shepherd Shamu, Moses J. Chimbari, Samson Mukaratirwa

**Affiliations:** 1 School of Nursing and Public Health, University of KwaZulu-Natal, South Africa; 2 Department of Community Medicine, Medical School, University of Zimbabwe, South Africa; 3 School of Life Sciences, University of KwaZulu-Natal, South Africa

## Abstract

**Background:**

Malaria is a serious public health problem in sub-Saharan Africa and is a leading cause of morbidity and mortality.

**Aim:**

To estimate the economic burden of malaria in rural households.

**Setting:**

The study was conducted in Gwanda district of Matabeleland South in Zimbabwe. A total of five malarious wards and all their households were selected for the study frame, out of which 80 households were chosen using clinic records.

**Methods:**

A retrospective analysis of secondary data and a cross-sectional household survey were conducted to estimate the household economic burden of malaria. Eighty households from five rural wards were identified from the health facility malaria registers and followed up. A household was eligible for inclusion if there had been at least one reported malaria case during the period of 2013−2015. Interviewer administered questionnaires were used to collect household data on economic costs of malaria.

**Results:**

Our findings showed that households spent an average of $3.22 and $56.60 for managing an uncomplicated and a complicated malaria episode respectively. A household lost an average of eight productive working days per each malaria episode resulting in an average loss of 24% of the monthly household income. An estimated 35%, mostly poorer households suffered catastrophic health expenditures.

**Conclusion:**

Malaria imposes significant economic burdens particularly on the poorer and vulnerable households. Although there are no user fees at rural clinics, households incur other costs to manage a malaria patient. These costs are far worse for complicated cases.

## Introduction

Malaria is a public health problem in sub-Saharan Africa, with an estimated 114 million people infected with malaria parasites in 2015. Africa accounted for 92% of the global malaria deaths in the same year.^1^ In addition to being a major health problem, malaria has a negative economic impact on socioeconomic development.^2,3,4^ Zimbabwe is one of the malaria endemic countries in Africa. However, the disease is only limited to specific malaria prone zones.^5^ Overall, malaria incidence in Zimbabwe has decreased over the past decade. However, it remains a major challenge in certain provinces, districts and wards.^6^ The economic costs of malaria place a burden on poorer and vulnerable households particularly in endemic regions.^7^ When malaria affects people in productive age groups, it increases their household expenses and at the same time reduces the productivity of the labour force.^8^ The costs of treatment of malaria as well as loss of productivity make up a large portion of the reduction in annual income and savings for the poorer households especially those that depend on farming for livelihoods^3,9^ and who may not have any other form of prepaid health insurance. Household costs include those spent on malaria prevention, treatment and related expenditure. Direct costs consist of costs for consultation, drugs, laboratory tests and transport to and from health facilities as well as subsistence costs during these trips.^10,11^ Indirect costs include time of productive work lost while caring for someone sick resulting in loss of income.^12^

The World Health Organization projected a 50–75% decrease in malaria incidence in Zimbabwe between 2000 and 2015.^13^ However, at a household level, data on the burden of those who are exposed to the disease and how they cope with the burden are scanty. Information on the household costs because of malaria provides policymakers with useful information on the impact of the disease on households. It is against this background that we carried out a study to determine the household economic costs because of malaria in Gwanda district, Matabeleland South Province of Zimbabwe.

## Research methods and design

### Study area and population

The study was carried out between October 2014 and May 2015. It was undertaken in five rural wards (Selonga, Sengezane, Ntalale, Buvuma and Nhwali) in Gwanda district located in Matabeleland South Province. The district lies midway between Zimbabwe’s second largest city of Bulawayo and the border town of Beitbridge. Gwanda lies in the natural regions IV and V, which are characterised by short rainfall seasons and incessant droughts.^14,15^ While Gwanda is not highly malarious, it has small pockets that are endemic, and these are often neglected or not given enough attention.

### Study design and data collection

This study used a mixed methods study design consisting of a retrospective records review and analysis, and a cross-sectional household survey of all households that had any malaria cases reported at the health facilities in the selected study wards and for the selected period of study. The records review was done to identify all reported and confirmed malaria cases from the malaria registers for the study period. Data collected from the records review included the patient’s age, sex, date of consultation and home address. All malaria cases were confirmed positive using rapid diagnostic tests (RDTs). Households were identified based on the information obtained from the malaria registers from the health facilities. The household survey was carried out in five wards namely Buvuma, Nhwali, Ntalale, Selonga and Sengezane. These wards were purposively selected because they had the highest number of reported malaria cases in the district. Each of the wards has one clinic. The inclusion criteria for a household were having at least one confirmed malaria case between 2013 and 2015 malaria seasons recorded at the health facility. Once selected, an interviewer administered household questionnaire was administered to the household head or their proxy. The questionnaires were administered by trained research assistants under the supervision of the principal investigator who checked all questionnaires for quality in the field. A household was defined as a unit consisting of a household head, children and related or unrelated persons who live together and constitute one unit^8^ with a combined income stream. Data collected from the household census included history of malaria cases in the households including hospitalisations, deaths, household assets, costs of managing a malaria episode and household productivity losses because of malaria. To determine costs because of malaria complication, respondents were asked about whether the patient was hospitalised or admitted to a hospital as a result of malaria. All malaria cases that resulted in hospitalisation were therefore assumed to be cases of complicated malaria as the data collected from the clinic records did not enable us to verify whether a case was complicated or not.

### Methodology for assessing household socioeconomic status

Principal Component Analysis (PCA) was used to construct household asset indices in order to determine the socioeconomic status of each household. Information on households’ ownership of commonly reported assets as used in demographic household surveys was used as input for the construction of the household asset indices. The household expenditure was also used as a complimentary method for assessing household living standard. The asset index and the expenditure living standard measures are considered to have less bias compared to household income. Using the asset index, the households were then grouped into five equal quintiles, with the first lower 20% of the households representing the lowest or poorest households and the fifth highest 20% households representing the highest or richest households. Household expenditure was used to assess the impact of health expenditures on household well-being. We categorised health expenditures as either catastrophic or non-catastrophic using threshold expenditures that were defined by Xu et al.^16^ They categorised household expenditures as catastrophic if out-of-pocket (OOP) health expenditures were equal to or exceeded 40% of the household’s non-subsistence consumption expenditure or capacity to pay.

Out-of-pocket health expenditures included payments made at the point of care, such as consultation fees, medication, hospital bills, laboratory costs and costs of alternative medicine or care. We modified Xu et al.’s^16^ household subsistence expenditure (SE) which defines a household’s SE as its average food expenditure (adjusted for household size) whose food share of total household expenditure lies between the 45th and 55th percentile. Instead, we used the average household food datum line for all Matabeleland South households as estimated by the country’s statistical body^17^ as a proxy measure. The estimated poverty datum line was US$1.22 per day and this was adjusted by household size to estimate each household’s SE.

### Data analysis

Data were entered in Microsoft Excel and then analysed with SPSS Statistics 22.0 (SPSS Inc., Chicago, IL) and Stata version 14 (StataCorp LP, College Station, TX). Descriptive statistics were used to examine the characteristics of the study sample. A Spearman’s correlation was run to assess the relationship between household size and number of malaria episodes. A two-sample *t*-test was used to determine the relationship between malaria cases and age as well as sex.

### Ethical considerations

The study was approved by the University of KwaZulu-Natal Biomedical Research Ethics Committee (Reference number BE 411/14) as well as the Medical Research Council of Zimbabwe (Reference number MRCZ/A/1870). Participation in this study was voluntary and all respondents who agreed to participate signed a written informed consent form after the objectives and procedures of the study had carefully been explained to them. Personal information from the participants was kept strictly confidential.

## Results

### Demographic analysis

**FIGURE 1:**
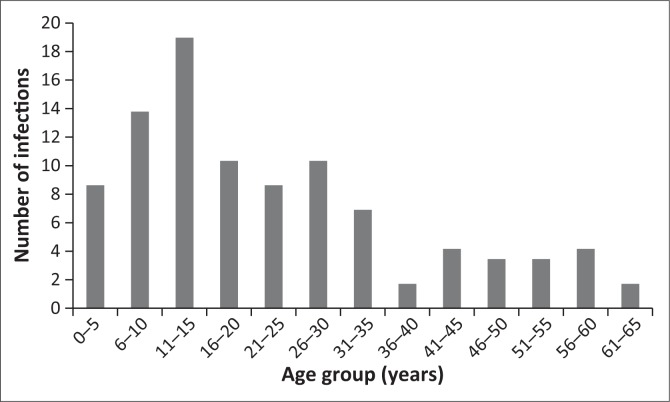
Age profile of malaria infected patients from households enrolled.

There were 109 malaria cases from 80 households recruited into this study. Some households reported more than one case of malaria over the study period. Fifty-six per cent of the respondents were male (Table 1). Most malaria patients were in the 11–15 years age group followed by the 6–10 years age group (Figure 1). Fifty-six per cent of malaria infections occurred in the older than 15 years age group.

**TABLE 1:** Distribution of respondents by selected demographic variables.

Selected demographic variable	Characteristics	*n* (%)
Sex	Male Female	45 (56.0) 35 (44.0)
Age	< 30 years 30 to < 60 years > 60 years	3 (3.8) 45 (56.2) 32 (40.0)
Family size	< 4 members 4 to 6 7 to 10 > 10	9 (11.3) 39 (48.8) 28 (35.0) 4 (5.0)
Employment status	Employed Not employed	16 (20.0) 64 (80.0)

*n*, number.

Male patients constituted 62.4% of the malaria cases. The majority (71.5%) of these cases were uncomplicated (Table 2). The two-sample *t*-test showed that uncomplicated cases of malaria were significantly associated with sex (*p* = 0.03). However, complicated cases of malaria were not significantly associated with sex. Children aged below 5 years made up 4.6% and 1.8% of uncomplicated and complicated cases of malaria, respectively. There was a significant association between malaria cases and the broad age groups (< 5 years and ≥ 5 years) for both uncomplicated (*p* = 0.008) and complicated (*p* < 0.001) malaria cases. The mean family size for a household was six people. There was a positive correlation between household size and number of malaria cases, which was statistically significant, Spearman’s correlation (*r*_s_) = 0.2494, *p* = 0.026.

**TABLE 2:** Distribution of malaria cases by sex, age and type of malaria case.

Sex	Total malaria cases	Uncomplicated cases			Complicated cases		
	*N* (%)	*n* (%)	% < 5 years	% ≥ 5 years	*n* (%)	% < 5 years	% ≥ 5 years
Males	68 (62.4)	54 (49.5)	3.7	46.8	18 (16.5)	1.8	13.8
Females	41 (37.6)	24 (22.0)	0.9	21.1	13 (11.9)	0.0	11.9

**Total**	**109 (100.0)**	**78 (71.5)**	**4.6**	**67.9**	**31 (28.4)**	**1.8**	**25.7**

*N*, number.

### Health outcomes

**FIGURE 2:**
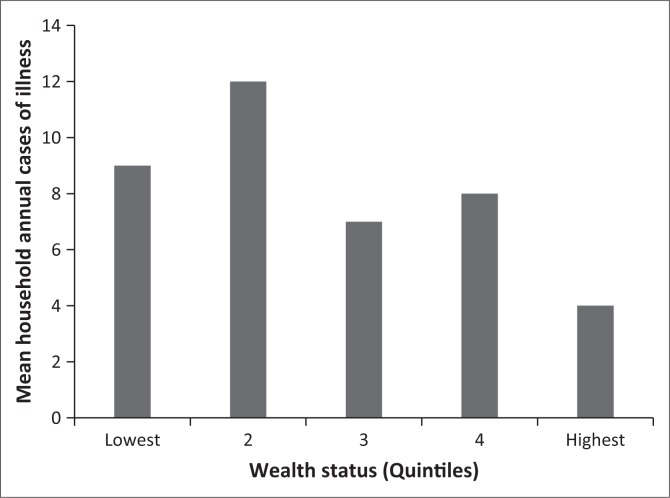
Distribution of mean household cases of malaria illness by wealth status.

**FIGURE 3:**
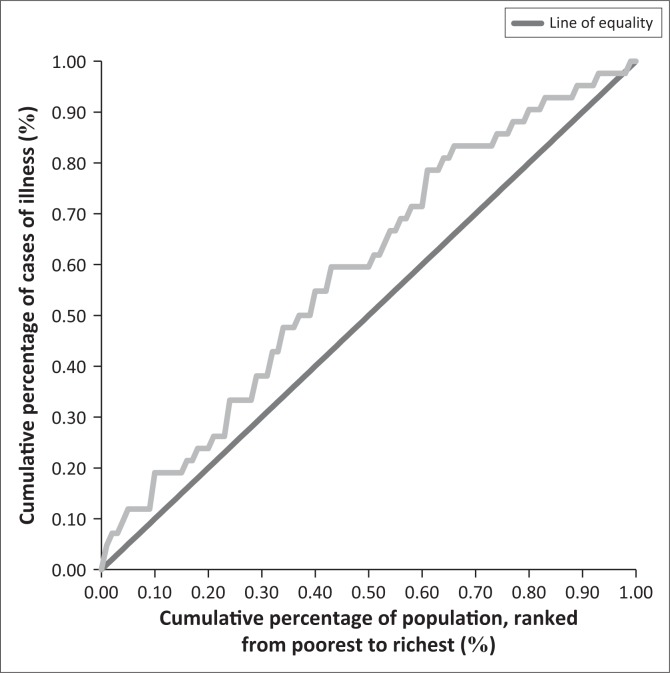
Concentration curve for annual cases of illness by wealth status.

The average annual malaria episodes were eight per household ranging from zero to a maximum of 36 cases per household. The distribution of the malaria episodes (Figure 2) shows that poorer households experienced the highest frequency of ill health. A concentration index and curve^18^ for the household’s cases of malaria illness showed that malaria cases were concentrated more among the poorer households. A curve that lies above the 45 degree line of equality has a negative concentration index and shows that the worse off were more affected (Figure 3). The differences in cases of malaria illness between the richer and poorer households were statistically significant (*p* < 0.05).

### Household expenditures on malaria

No household reported having any form of health insurance, which meant they relied on OOP heath expenditure. Free health services for malaria depended on the type of provider (public or private, church based or owned by the rural district council) and on the level of severity of the illness. Some facilities did not charge consultation fees, but charged for drugs, while others charged for both consultation and medication, and others did not charge for both services. Complicated cases referred to hospitals were charged. The average income per household was $118.60 per month, while the average expenditure was estimated at $110.20 per month, an average saving of about $8. The outpatient costs for treating an uncomplicated malaria case included transportation to and from the clinic for both the patient and those accompanying the patient, food and other complimentary costs of treatment such as nutritional foods and special diets for the patient and costs of alternative medicine or care. Inpatient costs for treating complicated cases of malaria included transport, consultation, diagnostic tests, drugs and bed charges. Mean monthly household expenditure on malaria was $19.87, which was about 17% of the mean household expenditure. Of the average monthly household direct and indirect general health expenditures of $24, malaria expenditures accounted for 83% of the expenditures. The main malaria expenditure item was OOP expenditures on inpatient costs for complicated malaria cases. However, payment for malaria treatment depended on the type of facility visited as mostly church related and rural district council run facilities charged some fees for both complicated and uncomplicated cases.

**FIGURE 4:**
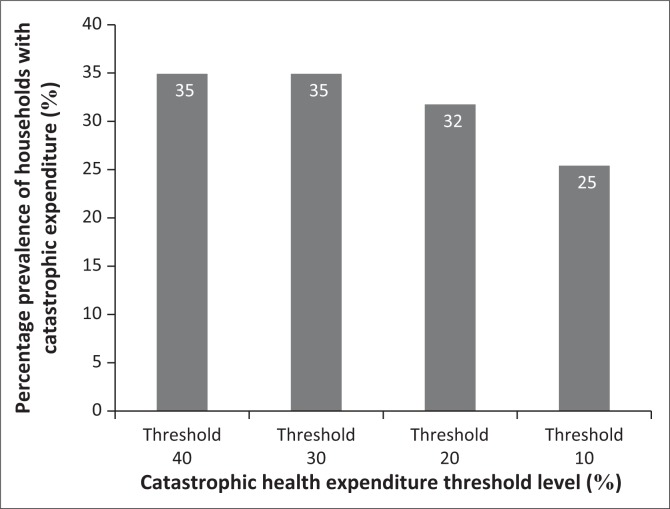
Percentage prevalence of households with catastrophic health expenditures at different thresholds.

The huge spread between the minimum and the maximum expenditures was as a result of the inclusion of both impatient and outpatient malaria related costs. Households spent a monthly average of $3.22 and $56.60 for managing an uncomplicated and a complicated malaria case, respectively. Of all the households that suffered catastrophic expenditures, 12.5% of them had had a member of the family hospitalised for more than 10 days per episode. Of the households that had malaria expenditures, 35% suffered catastrophic expenditures (Figure 4). While in this study we used 40% threshold, one can also use different thresholds to identify the number of households that fall into different categories for future monitoring of transition of households from one category to another.

### Loss of productive time because of malaria

Seventy per cent of respondents indicated that malaria sickness resulted in them failing to perform some productive activities such as working. About 63% of respondents reported failing to perform their normal household chores such as fetching water and firewood because of sickness from malaria. The number of productive days lost because of malaria ranged from 0 to 30 days. However, the average number of productive days lost because of sickness from malaria was eight days. The mean number of productive days lost while caring for a malaria patient in the household was seven days. The mean number of school days lost by a schoolgoing child because of malaria was eight days. Using the income approach and the national average minimum wage ($263.68)^19^ to assess productivity losses for the head of the household, in the absence of any labour substitution, households were likely to lose income of between $26.70 and $54.28 per malaria case. For caregivers, an equivalence rate of two person working days was used, which meant that a household that had a caregiver for malaria lost on average 3.3 equivalent days per malaria case, translating to a loss of income of between $13.35 and $27.14 per malaria case.

## Discussion

This study assessed the potential economic burden of malaria on households in Gwanda, Zimbabwe. Our findings showed that 56% of malaria infections occurred in the older than 15 age category. This age group is largely composed of the most productive individuals and therefore had a high indirect cost as a result of loss of productive time, confirming that malaria has a high economic impact in the area. Loss of productivity because of malaria is an important determinant of economic costs of the disease.^20^ The loss of an average of eight productive work days per malaria case showed that malaria has an economic impact on households by preventing people from carrying out their normal productive activities such as farming and rearing animals for each malaria case. Carers for malaria patients also lost seven days’ worth of wages while schoolgoing children lost on average, eight school days because of absenteeism from school. School absenteeism prevents normal educational development of the child and may have an impact on the family as the children have to make up the lessons they missed. In most instances, extra school lessons may have to be paid for, resulting in further impoverishment of households. The number of school days lost because of malaria illness in this study was twice as high as results from a study in Ghana.^21^ Reported cases of malaria in this study were at their peak during the rainy season when most agricultural activities took place. Households relying on agriculture as a source of income lost productive time either sick or caring for a sick person during a malaria episode.

No household reported having any form of health insurance, which meant they relied on OOP health expenditure. Malaria cases were categorised as complicated if there was hospital admission to manage the patient. This information was gathered from the respondents. Based on this information, the number of these complicated cases constituted 28% of all the malaria cases. Complications of malaria were assumed to be as a result of delays in seeking treatment at the health facility. The mean cost of treating a complicated case of malaria was very high ($56.60) compared to an uncomplicated case ($3.22). These costs were mostly through OOP payments. Our results for the cost burden for uncomplicated malaria were comparable to similar studies elsewhere.^10,22,23^ The high cost burden for complicated cases of malaria indicates the need for education of community members on the cost implications of late treatment of malaria as well as the possibilities of death occurring if treatment is not sought. Our findings show that those with a lower wealth status suffered more from malaria. This could be because households with a higher wealth status were able to afford malaria prevention products such as prophylactic drugs and mosquito repellents. Although the costs for managing an uncomplicated case were lower, households also bore a significant burden in terms of additional non-medical costs such as transport and food. Our data also showed that in some instances, malaria affected more than one household member per year, further increasing the household health expenditure and loss of productivity for poorer households. There was a positive correlation between household size and number of malaria cases, meaning that households with more family members were most likely to have more malaria cases. This has implications on household expenditure for treatment as this entails that there will be more costs for larger families. On average, poorer households spent proportionally more than richer households for malaria. This finding agrees with similar studies from Mozambique,^2^ South Africa^2^ and Kenya^11^ that had lower percentages. A survey in Malawi showed that expenditure on malaria by poor households was 32% of their annual income compared to only 4.7% for the high income households.^24^

Of the total number of malaria infections in this study, only 6.4% were from children below the 5 years age category. This could have been as a result of the malaria intervention programmes that target this age category as they are vulnerable to the effects of malaria. The number of malaria complications in this age category was only 1.8%. As transport costs constituted the bulk of the expenditure for managing an uncomplicated malaria case, policymakers should consider expanding the roles of community health workers to include provision of diagnosis and treatment of malaria. This has been shown to increase access to diagnosis and treatment, shorten clinical episode duration and reduce the number of severe cases.^25,26,27^ Easier access to health services will encourage affected communities to seek treatment early, resulting in a decrease in complications, thereby leading to reduced morbidity and mortality because of malaria.

This study showed that the costs of treating a complicated case of malaria were very significant and exerted an economic burden on households. There is therefore need for further studies to determine the health-seeking behaviour of communities in malaria endemic areas to find out more information and the reasons for delays in presenting at health facilities. We also recommend further research on the association between low wealth status and a higher number of malaria cases. The results from this study were not generalised to the whole district as only wards with the highest number of malaria cases in the district were selected.

## Conclusion

Malaria imposes significant economic burdens on rural households particularly the poorer and vulnerable households. The study showed that the economic burden of malaria resulted in some households incurring catastrophic health expenditures. Although there are no user fees at rural clinics for treatment of malaria, households incur other costs to manage a malaria patient. These costs are more for a complicated case of malaria as patients pay for drugs and other costs associated with hospital admission.
